# Beyond Technical Efficiency: Structural Disconnect Between Managerial Resource Use and Sustainability in Water Buffalo Farming in Türkiye

**DOI:** 10.3390/ani16050821

**Published:** 2026-03-06

**Authors:** Bekir Sıtkı Şirikçi

**Affiliations:** Department of Agricultural Economics, Faculty of Agriculture, Yozgat Bozok University, Yozgat 66900, Türkiye; b.sitki.sirikci@bozok.edu.tr

**Keywords:** water buffalo farming, technical efficiency, sustainability index, DEA, Tobit analysis, efficiency–sustainability nexus, SDG 2, Türkiye

## Abstract

Although higher technical efficiency is theoretically expected to enhance farm sustainability, empirical evidence in livestock systems remains ambiguous. This study investigates the interplay between technical efficiency and sustainability using data from 72 farms in Tokat, Türkiye, selected via stratified random sampling. Technical efficiency was calculated using Data Envelopment Analysis (DEA), while a multidimensional Sustainability Index was constructed using the analytic hierarchy process (AHP) for weighting dimensions. Determinants of inefficiency were estimated using a Tobit model. Results revealed an average technical efficiency of 0.717 and a Composite Sustainability Index of 0.41, classifying the sector as “moderate” but fragile. Crucially, the Kruskal–Wallis test showed no statistically significant difference in sustainability scores across efficiency groups (*p* > 0.05). This finding confirms a “structural disconnect,” demonstrating that high technical efficiency does not guarantee sustainability because of systemic bottlenecks such as dysfunctional organizations and infrastructure deficits. Furthermore, Tobit results showed that non-farm income and internet access were positively associated with technical efficiency, whereas indebtedness was negatively associated. Consequently, achieving lasting sustainability requires a shift from simple productivity support to structural modernization policies, including the integration of sustainability-oriented criteria such as institutional strengthening, environmental management, and financial capacity into existing support schemes.

## Simple Summary

This study examined the relationship between farm efficiency and sustainability in water buffalo farming using data from 72 farms in Tokat, Türkiye. Improving farm efficiency is generally expected to enhance the sustainability of livestock systems; however, empirical evidence in water buffalo production remains limited. Farm performance was evaluated based on how efficiently farmers managed and used available resources to produce output, while sustainability was assessed through economic, social, and environmental indicators. The results showed that the average efficiency level was moderate, whereas overall sustainability levels were relatively low, indicating a fragile production structure. Importantly, farms with higher efficiency were not necessarily more sustainable. This finding indicated a structural disconnect, meaning that improvements in efficiency did not automatically translate into better environmental, social, and economic outcomes. The analysis also showed that access to non-farm income and internet services was associated with better farm performance, whereas higher debt levels were linked to lower performance. The findings suggested that improving the long-term sustainability of water buffalo farming required not only higher efficiency but also broader structural improvements, including stronger farmer organizations, improved infrastructure, and better financial capacity. These insights may help policymakers design support programs that promote both efficiency and sustainability in livestock farming.

## 1. Introduction

**Figure 1:**
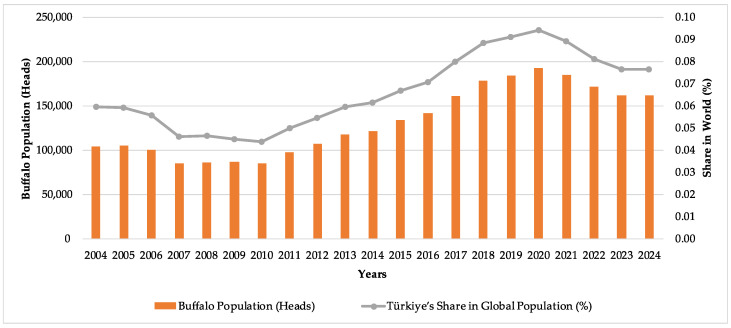
Temporal changes in the water buffalo population in Türkiye (2004–2024) and its proportional share within the global water buffalo population.

Water buffalo (*Bubalus bubalis*) production has been recognized as a production model that converts low-quality roughage into high-quality animal products, thanks to the species’ high resistance to climatic and climate-change-related temperature stress [1,2]. Amid increasing global population pressure, growing pressure on natural resources, and volatile market conditions, it was established that food security was determined not only by production volumes but also by the sustainability and resilience of production systems. In Türkiye, breeding projects and public support that have been implemented since 2010 have resulted in sectoral revitalization. The water buffalo population increased from 84,726 head in 2010 to 192,489 head in 2020 (an increase of 127%), and Türkiye’s share of the world water buffalo population rose from 0.04% to 0.09%, making Türkiye a production center both in Europe and globally [3]. However, as illustrated in Figure 1, the declining trend that began in 2020 led to the buffalo population falling to 162,051 head in 2024, suggesting that quantitative expansion alone may not be sufficient without structural sustainability. The contraction observed after 2020 coincides with a period characterized by the COVID-19 pandemic, increased macroeconomic volatility, and persistently high inflation in Türkiye. Since livestock support schemes are predominantly structured as per-head payments, the real value of these supports has eroded under inflationary pressures, thereby weakening their capacity to sustain herd expansion. In addition, compared to dairy cattle breeds, water buffalo exhibit relatively lower milk yield levels, which may limit their competitive position within the dairy market. These structural and economic factors collectively appear to have contributed to the slowdown and partial reversal of the growth trend observed during the previous decade. These findings suggest that transforming this biological and quantitative potential into a lasting economic model depends directly on technical knowledge at the farm level, managerial competence, and sustainable, highly resilient outcomes. In particular, the future of this sector, viewed as a resilient element of the traditional family-farm structure within rural household economies, warrants research into resource-use efficiency and productivity.

These developments highlight the importance of evaluating production performance not only in terms of quantitative expansion but also in terms of resource-use efficiency and long-term sustainability. In this context, technical efficiency provides a fundamental analytical lens for assessing how effectively production units transform inputs into outputs under existing technological conditions [4]. Numerous studies have examined efficiency measurement in the broader livestock sector [5,6,7,8,9,10,11,12,13,14,15,16,17,18]. However, within water buffalo production systems, empirical evidence remains comparatively limited [19,20,21,22,23,24,25,26].

In this context, socio-economic determinants of milk production in the Thanamalwila veterinary region of Sri Lanka were identified, and the technical efficiency of production was estimated [19]. The economic structure and efficiency of dairy water buffalo farms in the Çatalca district of Istanbul, Türkiye, were examined [20]. The economic efficiency of water buffalo farming conducted under the herdsman system in Indonesia was determined [21]. Production costs and technical efficiency levels of water buffalo milk farms in Iğdır Province, Türkiye, were determined [22]. The effect of cooperative membership on the technical and marketing efficiencies of water buffalo milk producers was investigated in the Philippines [23]. The efficiency of water buffalo farms operating under a semi-intensive system in Balıkesir Province was analyzed [24]. The efficiency scores of dairy water buffalo farms in the Philippine province of Nueva Ecija were measured [25]. The impact of development programs on water buffalo milk producers in the swampy regions of Iraq was evaluated, focusing on productivity levels and living standards [26].

Overall, these studies primarily emphasize input–output optimization and cross-sectional efficiency comparisons, while offering limited evidence on whether efficiency differentials translate into multidimensional sustainability outcomes in water buffalo farming.

Against this background, sustainability was presented as a broader evaluative perspective for livestock systems that extended beyond productivity performance. In a broad sense, it was considered to encompass economic, social, and environmental dimensions. Economic sustainability was associated with the ability of production systems to remain viable and resilient over time; social sustainability was linked to the continuity of rural livelihoods, social structures, and community well-being; and environmental sustainability was defined in relation to the responsible use and preservation of natural resources in ways that did not compromise ecological balance in the long run.

From a broader sustainability perspective, the sustainability of livestock systems has been analyzed in the literature using multidimensional methodologies that include economic, social, and environmental dimensions, as well as institutional and technological components, in accordance with regional dynamics [27,28,29,30,31,32,33,34,35,36,37,38,39,40,41,42,43,44]. In this context, integrated assessment methods were developed for small-scale dairy farms in India [27]; the sustainability status of cattle breeding in Indonesia was examined using Multidimensional Scaling (MDS) [28]; and the sustainability of cattle breeding was evaluated through key indicators [29]. Within the framework of geographical and ecosystem-focused approaches, indicators were produced using participatory rural assessment techniques in South Africa [30], and fuzzy logic-based decision support systems were defined in the Pantanal wetlands of Brazil [31]. In studies that employed greater methodological diversity, the sustainability status of dairy farming areas was analyzed [32], conventional and organic production units were compared using the MESMIS methodology [33], and general frameworks for sustainability indices of integrated facilities were presented [34]. Integrated sustainability indicators for milk production systems were proposed in Colombia [35], and comprehensive scoring methods based on scientific information were developed for beef production systems [36]. In European and Latin American examples, researchers compared extensive farms in Spain according to their organic orientation [37], analyzed silvopastoral systems in Mexico using the SAFA framework, and developed unique indicators for production models in the Brazilian Amazon [38,39]. Studies conducted in Türkiye characterized the heterogeneity of water buffalo breeding systems in the Marmara Region [40] and determined the effect of farm size on the sustainability of beef cattle farms in Samsun Province [41]. Recent research re-evaluated cattle production systems within the MESMIS framework [42], designed nonlinear regression models to measure rural sustainability [43], and analyzed cattle integration using the RAP-Integration approach [44].

Although sustainability has been widely examined in livestock systems using multidimensional assessment frameworks, empirical studies explicitly measuring sustainability performance in water buffalo production remain relatively limited. Moreover, existing research rarely integrates technical efficiency analysis with multidimensional sustainability assessment within a unified empirical framework. While technical efficiency is generally associated with improved resource allocation and is often considered a necessary condition for sustainable production, it does not automatically ensure balanced outcomes across economic, social, and environmental sustainability dimensions. In this sense, efficiency and sustainability can be viewed as analytically related yet structurally distinct constructs that may reinforce—but do not inevitably guarantee—one another.

Against this background, the relationship between farms’ technical efficiency (operational performance) and their multidimensional sustainability outcomes remains insufficiently understood, particularly with respect to whether efficiency gains are consistently reflected across economic, social, and environmental dimensions.

Rather than presuming a direct correspondence between efficiency gains and sustainability outcomes, this study explores whether a structural disconnection may exist between productivity-oriented performance and multidimensional sustainability achievements in water buffalo farming.

Accordingly, the primary objective of this study was to assess the technical efficiency levels of water buffalo farms using Data Envelopment Analysis and to evaluate their multidimensional sustainability performance through a composite index framework. In addition, it was examined whether farms with different efficiency levels exhibited systematic differences in economic, social, and environmental sustainability outcomes. Furthermore, the structural and socio-economic determinants of inefficiency were identified using a Tobit model in order to better understand the factors shaping performance variation within the Turkish buffalo production context. The analysis was conducted using farm-level data from Türkiye, thereby providing insights relevant to similar low-input livestock systems.

## 2. Materials and Methods

### 2.1. Study Area

According to 2025 data from the Turkish Statistical Institute, Tokat accounted for 4.92% of the national water buffalo population and ranked sixth nationwide. Over the past decade, its ranking fluctuated between third and sixth place. National statistics further showed that buffalo production in Türkiye was concentrated in a limited number of provinces, while the population shares of other provinces remained comparatively lower [45].

Tokat’s agro-ecological characteristics—particularly the Yeşilırmak Basin and the Kazova Plain—provide favorable conditions for pasture-based buffalo production, supported by suitable climatic conditions and water availability. In addition to its quantitative significance at the national level, the selection of the research area was influenced by these structural and production-related characteristics, especially its low-cost, pasture-based feeding system. These features made Tokat a relevant case for examining the efficiency and sustainability dynamics of buffalo farming systems.

### 2.2. Data Collection

In determining the sample size, the stratified random sampling method was preferred to increase the representativeness of the sample relative to the population and to minimize sampling variance [46]. Since the farms in the research area did not exhibit a homogeneous distribution of water buffalo, they were divided into homogeneous strata based on population size. The sample size was calculated to be 72 farms at a 99% confidence level and a 10% margin of error (Equation (1)). The Neyman method was used to allocate the determined sample size across strata (Equation (2)) [47].

(1) $$n=\frac{{\left(N_{h}\;\times \;S_{h}\right)}^{2}}{N^{2}\;\times \;D^{2}\sum N_{h}\;\times {{\;S}_{h}}^{2}}\;$$

(2) $$\mathrm{nh}=\frac{N_{h}\;\times {\;S}_{h}}{\sum N_{h}\;\times \;S_{h}}\;\times \;n\;$$

n: sample size;

N: total number of units in the population;

Nh: number of units in the h-th stratum;

Sh^2^: variance of the h-th stratum;

D^2^ = (d^2^/z^2^) statistical precision factor.

Fieldwork and face-to-face interviews with selected farmers were conducted in February 2025. The collected data included detailed input–output records, financial costs, and production activities for the production season from February 2024 to January 2025.

Before commencing the data collection process, the necessary permission was obtained from the Yozgat Bozok University Social and Human Sciences Ethics Committee (Decision No: 21/44). The research process was conducted in full compliance with the ethical principles outlined in the Declaration of Helsinki. Following data entry, the raw data were preprocessed to identify outliers and missing values and prepared for statistical analysis. Prior to empirical modeling, the content validity of the questionnaire was assessed during the design phase through a comprehensive review of the relevant literature and pilot tests. The draft questionnaire was refined to improve clarity, coherence, and conceptual alignment before the final survey was administered.

Following data collection, the internal consistency of the multi-item constructs was assessed using Cronbach’s alpha based on the original Likert-scale responses, indicating acceptable to high reliability levels.

### 2.3. Data Analysis

Data obtained from the field study were analyzed using a four-stage methodological framework consisting of efficiency measurement, index creation, statistical comparison, and regression analysis.

#### 2.3.1. Technical Efficiency Analysis (Data Envelopment Analysis)

In this study, an input-oriented Data Envelopment Analysis model, a nonparametric frontier approach assuming variable returns to scale (VRS), was applied to quantitatively determine the technical efficiency of water buffalo breeding farms. Technical efficiency, a common measure of technical rationality in agricultural production, was defined as the extent to which farms successfully transform their inputs into outputs using their existing production capabilities [4].

Studies focusing on water buffalo milk production and farm performance found that researchers using Data Envelopment Analysis preferred different input- or output-oriented models and variable sets, depending on the conditions of the study area and the data structure. In another study analyzing farms in Çatalca, Istanbul, both input- and output-oriented models were used; feed, labor, veterinary health, depreciation, and electricity and water costs were included as inputs, while milk income, calf income, and income from increases in inventory value were included as outputs [20]. In a study conducted on farms in Iğdır province, an input-oriented approach was preferred to achieve resource savings [22]. In this analysis, amounts of roughage and concentrate feed, labor, number of milking water buffaloes, veterinary medicine costs, other costs, and barn size were used as inputs, and the total annual milk production was used as the output. In a study examining farms in Balıkesir Province, Türkiye, researchers adopted an output-oriented model because the aim was to maximize outputs by improving production techniques [24]. In this context, the number of animals, feed, labor, veterinary costs, and other variable costs were considered inputs, and gross production value, which includes milk, cream, meat, fertilizer, and increases in inventory value, was considered the output variable. Similarly, in a study analyzing farms in Nueva Ecija, Philippines, an input-oriented model was adopted: the quantities and costs of biological materials (vitamins, parasite medication), feeds, roughage, and labor were determined as inputs, and milk production volume and value were determined as outputs [25].

This study, based on the assumption that water buffalo breeding farms did not operate at their optimal production scale and on the consideration that farms were better able to control inputs than to increase output because of market conditions or biological limitations, selected an input-oriented DEA model to estimate potential input savings while maintaining the current output level. Within the model specifications, one output variable and five input variables were used:

Output: Gross production value (GPV) ($ farm^−1^) was obtained from water buffalo breeding.

Inputs: (1) concentrate feed (kg dry matter^−1^), (2) roughage (kg dry matter^−1^), (3) water buffalo population (livestock units), (4) labor utilization (male labor unit), and (5) veterinary health expenditures ($).

All monetary variables were converted from Turkish Lira (TRY) to U.S. dollars (USD) using the official average USD/TRY exchange rate for the period February 2024–January 2025 (1 USD = 35.37 TRY), as published by the Central Bank of the Republic of Türkiye [48].

The DEAP (version 2.1) software package, developed by Coelli and widely used in the literature, was used to conduct efficiency analyses [49]. The software was used to calculate the efficiency scores of decision units and to determine the boundary values [50].

#### 2.3.2. Measuring Sustainability

In this study, the Composite Sustainability Index (CSI) method was used; it addressed sustainability across its dimensions and allowed the development of indicators specific to the research topic in line with the stated objective. Studies in the literature that analyze the performance of livestock farms using similar composite-indexing methods are methodologically consistent with this study. In a study on cattle breeding in Indonesia, sustainability was scored using the multidimensional scaling (MDS) technique across ecological, economic, social, technological, and institutional dimensions and converted into an index ranging from 0 to 100% [28]. Similarly, a sustainability index was calculated using the Rap-SIBUSAPE approach to determine the current situation and future projections in dairy farming [32]. In a study conducted in India, the Composite Sustainability Index (CSI) method was applied, in which economic, social, and environmental indicators were standardized using normalization formulas [29]. In a comprehensive study analyzing the sustainability of native cattle breeds, the economic, social, and environmental dimensions were normalized using the min–max method and analyzed using the Sustainability Index (SI) model [51]. In a study of beef farms in Türkiye, numerous social and environmental indicators were grouped via factor analysis, and economic efficiency was estimated using DEA to derive a holistic index [41].

The Sustainability Index was constructed through a series of logical steps: selection of dimensions and variables, data transformation (normalization), weighting, and evaluation (using threshold values). The current state of sustainability in water buffalo breeding was presented holistically, encompassing economic, social, and environmental dimensions.

The structural characteristics of water buffalo breeding and the methodological frameworks commonly accepted in the literature served as the basis for creating the indicator sets. Studies that holistically address sustainability on livestock farms were used to guide the selection and sizing of variables [27,28,29,30,33,35,37,39,40,41,42,51,52,53].

The sustainability indicators evaluated in this analysis were selected to holistically reflect the technical, social, and ecological performance of the farms; the positive (+) or negative (−) impact of each variable on sustainability was predefined. In this context, economic sustainability indicators reflecting the financial resilience, productivity, and management capacity of the farms are presented in Table 1.

Social sustainability parameters, which determine the human capital structure and the level of social integration, are detailed in Table 2.

Environmental sustainability indicators, encompassing the use of natural resources and environmentally friendly infrastructure, are presented as an integrated set in Table 3. These indicator sets were designed to reflect both the unique characteristics of the local water buffalo production system and internationally accepted sustainability criteria.

To make the indicators with different units comparable and summable, the min–max normalization method was applied, and all values were standardized to the range of 0–1. During normalization, the following equations were used according to the direction of each indicator’s impact on sustainability (Equations (3) and (4)) [54]:

(3) $$l_{\mathrm{ij}}\;=\;\frac{X_{\mathrm{ij}}-\mathrm{Min}X_{\mathrm{ij}}}{\mathrm{Max}X_{\mathrm{ij}}-\mathrm{Min}X_{\mathrm{ij}}}\;$$

(4) $$l_{\mathrm{ij}}=\frac{\mathrm{Max}X_{\mathrm{ij}}-X_{\mathrm{ij}}}{\mathrm{Max}X_{\mathrm{ij}}-\mathrm{Min}X_{\mathrm{ij}}}$$

In this formula, i represents the indicator numbers 1, 2, 3… n; j represents the sustainability indicators; and X_ij_ denotes the values of the indicators.

In multidimensional constructs such as sustainability—where economic, social, and environmental dimensions comprise heterogeneous indicators—the determination of indicator-level weights is methodologically complex. Various approaches have been proposed, including equal weighting, AHP, PCA, benefit-of-the-doubt methods, and regression-based techniques; however, no universally accepted weighting scheme exists, and each involves normative assumptions [55]. In this study, equal weighting was applied at the indicator level within each sustainability dimension. This choice was guided by three considerations: (i) the indicators were selected based on theoretical coherence and prior empirical applications in livestock sustainability research; (ii) equal weighting minimizes additional subjectivity when empirical justification for differential importance is limited; and (iii) similar farm sustainability studies have adopted simple averaging when indicators represent conceptually related constructs [29,41,51]. Accordingly, each indicator was assumed to contribute equally to its respective dimension, and dimension scores were calculated using arithmetic means.

The basic mathematical approach used in the calculation is given by Equation (5) below. While indicator weights were equal within each dimension, the relative weights of the three sustainability dimensions were subsequently derived using the analytical hierarchy process (AHP).

(5) $$\mathrm{ESI}=\frac{\sum_{i}^{n}\mathrm{lij}}{n}\;\;\;\;\;\;\mathrm{SSI}=\frac{\sum_{i}^{n}\mathrm{lij}}{n}\;\;\;\;\;\;En\mathrm{SI}=\frac{\sum_{i}^{n}\mathrm{lij}}{n}$$

ESI = Economic Sustainability Index;

SSI = Social Sustainability Index;

EnSI = Environmental Sustainability Index;

n = indicator number;

lij = indicator values.

After calculating the economic, social, and environmental sustainability dimension indices using Equation (5), a composite indexing procedure was applied to determine the overall sustainability scores of the farms.

Since sustainability is inherently a multidimensional concept and the relative importance of its components cannot be assumed to be equal, an objective and theoretically grounded weighting framework was required. In this context, the analytical hierarchy process was adopted, as comparable multi-criteria weighting approaches have been applied in livestock sustainability and agricultural performance assessment studies [35,56,57].

The AHP method was originally developed by Saaty (1980) as a structured yet flexible multi-criteria decision-making technique that allows complex evaluation problems to be decomposed into a hierarchical structure and facilitates pairwise comparisons among criteria, thereby ensuring internal consistency within the weighting scheme [58].

The weights of the three sustainability dimensions included in the Composite Sustainability Index (CSI)—ESI, EnSI, and SSI—were determined using expert evaluations obtained from a group of 17 specialists, each with more than 10 years of professional experience in agricultural sustainability. The group consisted of academics, a leading buffalo farmer, a veterinarian, and an agricultural engineer, thereby ensuring that scientific knowledge, practical field experience, and institutional perspectives were collectively reflected in the weighting process.

Pairwise comparisons were conducted using Saaty’s fundamental 1–9 scale, where 1 denotes equal importance and 9 denotes extreme importance of one criterion over another [59]. Individual expert judgments were aggregated using the geometric mean, as shown in Equation (6) [60].

(6) $${\overset{-}{a}}_{ij}={\left(\prod_{k=1}^{17}a_{ij}^{\left(k\right)}\right)}^{\frac{1}{17}}$$

The aggregated elements obtained from Equation (6) were then used to construct the aggregated pairwise comparison matrix A presented in Equation (7).

(7) $$A=\left[\begin{array}{ccc} 1 & 2 & 2\\ 0.5 & 1 & 2\\ 0.5 & 0.5 & 1 \end{array}\right]$$

The priority weight vector was derived from the aggregated comparison matrix using the normalized principal eigenvector approach (row-average approximation), yielding the weights reported in Equation (8).

(8) $$w=\left[\begin{array}{c} w_{ESI}\\ w_{EnSI}\\ w_{SSI} \end{array}\right]=\left[\begin{array}{c} 0.49\\ 0.31\\ 0.20 \end{array}\right]$$

To assess the internal consistency of the aggregated judgments, the maximum eigenvalue $\lambda_{max}$ was computed and used to calculate the Consistency Index (*CI*), as defined in Equation (9). For n=3 and RI=0.58, the Consistency Ratio (*CR*) was calculated according to Equation (10).

(9) $$CI=\frac{\lambda_{max}-1}{n-1}$$

(10) $$CR=\frac{CI}{RI}\approx 0.034$$

Since CR<0.10, the aggregated expert evaluations were considered sufficiently consistent [61].

The AHP-derived weights (Equation (8)) were subsequently incorporated into the composite aggregation framework to compute the overall Composite Sustainability Index (CSI). The CSI was calculated as a weighted linear combination of ESI, EnSI, and SSI, presented in Equation (11).

(11) CSI=0.49∗ESI+0.31∗EnSI+0.20∗SSI

Sustainability thresholds were defined following [52], and the results were classified as “Unacceptable” (0.00–0.20), “Borderline” (0.21–0.40), “Average” (0.41–0.60), “Good” (0.61–0.80), and “Best” (0.81–1.00).

#### 2.3.3. Statistical Analysis of Efficiency Groups

Based on the calculated efficiency scores, farms were classified into three groups: inefficient (≤0.50), moderately efficient (0.51–0.74), and efficient (≥0.75). The normality of sustainability scores within each efficiency group was assessed using the Shapiro–Wilk test. For environmental sustainability, deviations from normality were observed in the moderately efficient group (W = 0.869, *p* = 0.011) and the efficient group (W = 0.905, *p* = 0.009). For economic sustainability, normality was rejected in the inefficient group (W = 0.837, *p* = 0.003). For composite sustainability, deviations from normality were detected in all three groups (inefficient: W = 0.796, *p* < 0.001; moderately efficient: W = 0.746, *p* < 0.001; efficient: W = 0.898, *p* = 0.006). In contrast, social sustainability scores did not significantly deviate from normality in any efficiency group (inefficient: W = 0.925, *p* = 0.109; moderately efficient: W = 0.973, *p* = 0.821; efficient: W = 0.981, *p* = 0.850). Given the partial violation of the normality assumption and to ensure methodological consistency across sustainability dimensions, the nonparametric Kruskal–Wallis H test was employed to assess differences in sustainability performance among the efficiency groups. The Shapiro–Wilk normality test and the Kruskal–Wallis H test were conducted using Jamovi statistical software (Version 2.3).

#### 2.3.4. Determinants of Inefficiency (Tobit Regression Model)

In the final stage, socio-economic factors that cause technical inefficiency on farms were analyzed. Since the efficiency scores (the dependent variable) were censored between 0 and 1, the Tobit regression model was used to obtain consistent estimators [62]. Given that DEA-based efficiency scores were bounded between 0 and 1 and exhibit right-censoring at the upper limit (efficiency score = 1), the Tobit model provided consistent maximum likelihood estimates under censored data conditions. Unlike OLS, which assumes an unbounded dependent variable, the Tobit specification explicitly accounts for censoring. Furthermore, the objective of this stage was to identify determinants of inefficiency rather than re-estimate the production frontier, and the literature indicates that the Tobit model has been widely used to examine variables affecting the performance of cattle and water buffalo farms because of the limited structure of the data. Within this scope, studies have reported that producer demographics (age, education, experience), farm characteristics (scale, ownership, geographic location), breeding practices, socio-economic variables, and indicators representing the local policy environment were included as explanatory variables in the model [5,17]. Similarly, Tobit regression analysis was used in research aimed at identifying determinants of water buffalo farm efficiency in Türkiye [24]. Equation (12) [63] defines the linear relationship between the independent variables and the latent variable in the Tobit model:

(12) $$y_{i}^{*}=X_{i}\beta +\varepsilon_{i}$$

Here, $X_{i}$ represents the vector of explanatory variables, β represents the parameter vector of the estimated coefficients, and $\varepsilon_{i}$, represents the error term. The basic mathematical framework of the Tobit model is presented with Equation (13) [64]:

(13) $$y_{i}=\left\{\begin{array}{c} y_{i}^{*}\;\;\;\;\;\;if\;0\;<y_{i}^{*}<1\\ \;\;\;\;\;\;\;\;1\;if\;\;y_{i}^{*}\;\geq 1\\ \;\;\;\;\;\;\;\;0\;if\;\;y_{i}^{*}\;\leq 0 \end{array}\right.$$

Here, the first term represents the observed efficiency score, and the second term represents the unobservable variable $\;y_{i}y_{i}^{*}$. In the Tobit model designed to analyze factors determining farms’ technical inefficiency in this study, variables related to farm manager demographics, breeding experience, and the farm’s economic structure were used, consistent with the literature. Technical efficiency scores calculated by DEA for each farm served as the model’s dependent variable. The independent variables included demographic indicators, such as education level, as well as variables representing non-agricultural income, general experience in livestock and plant production, debt status, and internet connection (representing access to technology). Several alternative model specifications were estimated, and model selection was guided by information criteria (AIC and BIC). The specification with the lowest AIC value was retained as the final model to ensure an appropriate balance between goodness-of-fit and model parsimony. Average marginal effects (AME) were calculated based on the Tobit specification to assess the marginal impact of explanatory variables on the observed (censored) efficiency scores. Diagnostic tests were conducted to assess model adequacy. The White test was applied to examine heteroskedasticity, and variance inflation factors (VIF) were used to evaluate potential multicollinearity among the explanatory variables. The Tobit regression analysis was performed using EViews 13. 

## 3. Results

Descriptive statistics for the farms included in the study are summarized in Table 4. Gross value of production (GPV) was used as the output variable in the efficiency analysis, while roughage, concentrate feed, Livestock Units (AU), labor, and veterinary costs were used as input variables. According to the results, the average GPV per farm was $13,800.57. However, production values across farms varied between $3015.19 and $52,692.72, indicating substantial variability. Examination of input usage levels revealed significant differences among farms, particularly in feeding practices. The average roughage usage per farm was 39,833.97 kg, and the average concentrate feed usage per farm was 13,151.11 kg. The standard deviations of the input variables were found to be considerably higher than their means, indicating heterogeneity in the regional production system. The minimum values for concentrate feed use (0.01 kg) and labor input (34.5 h) were observed to be substantially lower than the corresponding sample averages. Considerable variation across farms was detected, particularly with respect to pasture utilization levels. Farm sizes were standardized using LU coefficients, and the average farm size was determined to be 22.37 LU. Farm sizes ranged from 4.50 LU to 83.56 LU. In water buffalo breeding, an average of 1614.25 h of labor was used per farm, while the amount spent on veterinary services was approximately $509.97.

The frequency distribution and summary statistics of the technical efficiency (TE) scores of water buffalo farms in the research area are presented in Table 5. According to the DEA results, the farms’ average technical efficiency score was 0.717. This value indicated that, under current production technologies and management conditions, farms could reduce input usage by an average of 28.3% without changing output levels.

The distribution of efficiency scores ranged from a minimum of 0.221 to a maximum of 1.000. Out of the 72 farms analyzed, 14 (19.4%) were found to be technically efficient (efficiency score = 1.000). The high standard deviation (0.236) indicated substantial disparities between farms in managerial success and resource-utilization skills.

The frequency distribution indicated that 43.06% (31 sampled farms) had high efficiency scores (0.75–1.00). In contrast, 29.17% (21 farms) performed poorly, falling below 0.50. The percentage of farms showing moderate efficiency (0.51–0.74) was 27.78% (20 farms). The results revealed that a significant proportion of farms in the region required radical improvements in their production processes to catch up with best-practice peers.

The economic sustainability indices of the farms included in the research and the findings related to the sub-components of these indices are summarized in Table 6, classified according to their technical efficiency levels. Based on the calculations, the average Economic Sustainability Index across all farms was 0.45.

When the technical efficiency groups were examined, the Economic Sustainability Index was 0.43 in the “inefficient” group, increased to 0.47 in the “moderately efficient” group, and reached 0.46 in the “efficient” group. However, the Kruskal–Wallis test indicated that the observed fluctuations between groups were not statistically significant (*p* = 0.276).

Upon detailed examination of the sub-components, the “relative profit” indicator was 0.40 in efficient farms, compared to 0.16 and 0.29 in the other groups, respectively. In contrast, the “income–expense record-keeping” indicator was 0.10 in the efficient group. The production cost index was calculated as 0.75 in the moderately efficient group and 0.71 in the efficient group. The “forage crop production” index was 0.90 across all groups, regardless of the technical efficiency level.

The levels of social sustainability of farms in the research area and the sub-parameters comprising this index were analyzed across technical efficiency groups and summarized in Table 7. Based on the calculations, the region’s average Social Sustainability Index was 0.44.

When examined by technical efficiency groups, the Social Sustainability Index was 0.43 for inefficient and moderately efficient farms and 0.45 for efficient farms. However, the Kruskal–Wallis test revealed that this observed difference between groups was not statistically significant (*p* = 0.304).

Detailed analysis of the sub-indicators showed that the highest score was observed in “access to veterinary and other health services” (0.99), followed by “agricultural organization” and “the importance of the family idea in the decision-making process for water buffalo breeding,” both with scores of 0.94. However, a striking paradox was observed: while the level of “agricultural organization” was 1.00 (full participation) in efficient farms, the level of “participation in agricultural extension activities” was 0.00 in the same group.

The weakest links negatively affecting social sustainability were agricultural extension activities (0.04) and social activities (0.07). Furthermore, the “satisfaction with social life” level remained at 0.17 in the overall average.

One of the largest numerical differences between technical efficiency groups was observed for access to infrastructure, although this difference was not statistically significant. The adequacy of transportation and infrastructure services was measured at 0.67 for inefficient farms and 0.84 for efficient farms.

Table 8 presents the environmental sustainability levels of farms in the research area and the index scores of the sub-indicators comprising this dimension. According to the results, the overall Environmental Sustainability Index averaged 0.50 points. When technical efficiency groups were examined, the Environmental Sustainability Index score increased from 0.47 in inefficient farms to 0.53 in efficient farms, with 0.49 in moderately efficient farms. The Kruskal–Wallis test indicated no statistically significant differences (*p* = 0.341).

When the environmental indicators were examined in detail, a substantial gap was observed between the farmers’ perceived awareness indices and their implementation indices. Farms were found to have index scores close to the maximum (1.00) in the indicators of “paying attention to environmentally friendly practices in cultivation” (0.97) and “paying attention to the hygiene of tools and equipment” (0.99). However, the “presence of manure pits” index remained extremely low, with an overall average of 0.15.

The most significant proportional divergence between the efficiency groups was observed in the infrastructure indices. The manure pit presence index was 0.05 in inefficient farms and increased to 0.29 in efficient farms. Similarly, the perception index regarding “sufficiency of pasture areas” reached 0.52 points in efficient farms, compared to 0.38 points in inefficient farms. The indicator “Considering livestock farming important for biodiversity” was reflected in a score of 0.78. However, the “covered space per animal” index was measured at 0.08.

The results of the Composite Sustainability Index (CSI), which integrates economic, social, and environmental dimensions within a holistic framework, were presented in Table 9. Based on the calculations, the Composite Sustainability Index of water buffalo-breeding farms in the research area averaged 0.41. According to the reference ranges defined in the Methodology section, this value corresponded to the “moderate” sustainability level (0.41–0.60).

A comparison of sub-dimensions indicated that the environmental dimension contributed the highest score (0.50), followed by the economic dimension (0.45). The lowest contribution was observed in the social sustainability dimension, with a score of 0.44.

When the effect of technical efficiency on sustainability performance was examined using the Kruskal–Wallis test, no statistically significant differences were identified between efficiency groups in any dimension, including the Composite Sustainability Index (*p* = 0.103), economic (*p* = 0.276), social (*p* = 0.304), and environmental (*p* = 0.341) dimensions. The Kruskal–Wallis test results are presented in Table 10.

**Figure 2:**
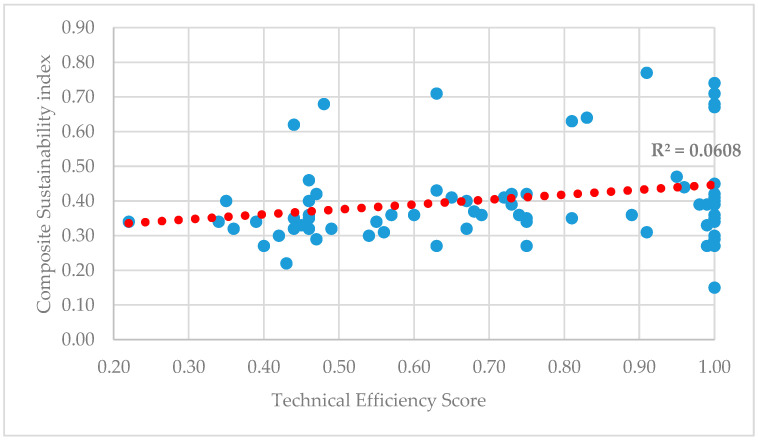
Relationship between technical efficiency scores and the Sustainability Index.

The scatter plot illustrating the relationship between farms’ technical efficiency scores and composite sustainability index scores was presented in Figure 2. The distribution of the data points exhibited a dispersed pattern rather than a clear linear association, and the fitted regression line appeared approximately horizontal.

The calculated coefficient of determination (R^2^ ≈ 0.06) indicated that variations in technical efficiency accounted for only a very small proportion of the variation in sustainability performance. This simple linear regression was presented for descriptive and exploratory purposes to visualize the pattern of association between the two variables.

The primary inferential assessment of differences in sustainability performance across technical efficiency groups was conducted using the Kruskal–Wallis test. Consistent with these test results, no statistically significant relationship was identified between technical efficiency and composite sustainability.

Descriptive statistics for the variables used in the Tobit model were presented in Table 11. Farmers had an average of 32.75 years of experience in crop production and 32.46 years of experience in livestock breeding.

The mean education level was 2.56, corresponding approximately to lower-to-upper secondary education.

Regarding economic characteristics, 48.6% of the farmers reported having non-farm income, while 70.8% were classified as indebted.

In terms of digital infrastructure, 62.5% of the farms had access to a computer, and 75.0% reported having an internet connection.

Table 12 presents the Tobit regression estimates and the corresponding average marginal effects (AME) for the determinants of technical efficiency in water buffalo breeding farms. The likelihood ratio statistic indicated that the model was jointly significant (LR χ^2^(7) = 29.23, *p* < 0.01), confirming that the explanatory variables jointly explain variations in efficiency. The presence of right-censoring (14 out of 72 observations) supported the appropriateness of the Tobit specification. Diagnostic tests indicated no multicollinearity problem, as all centered variance inflation factors (VIFs) were below the conventional threshold of 5. The White heteroskedasticity test failed to reject the null hypothesis of homoskedasticity (*p* > 0.05), indicating no evidence of heteroskedasticity.

Experience in plant production was negatively and statistically significantly associated with technical efficiency at the 1% level (AME = −0.011). This result indicated that an additional year of crop production experience was associated with a 0.011 decrease in the efficiency score.

Non-farm income was positively associated with technical efficiency and was statistically significant at the 1% level. The average marginal effect (0.206) suggested that farms with alternative income sources were associated with, on average, a 0.206-point higher efficiency score compared to those without non-farm income.

Experience in livestock breeding was positively and statistically significantly associated with technical efficiency (AME = 0.013, *p* < 0.01). Each additional year of livestock experience was associated with an approximately 0.013-point higher efficiency score.

Indebtedness status was negatively associated with technical efficiency and was statistically significant at the 1% level (AME = −0.164). Farms with outstanding debt exhibited lower efficiency levels relative to non-indebted farms.

With regard to digital variables, internet access exhibited a positive and statistically significant association with technical efficiency at the 5% level (AME = 0.142). This finding suggested that farms with internet access were associated with, on average, a 0.142-point higher efficiency score compared to those without internet connectivity.

In contrast, computer ownership did not show a statistically significant association with technical efficiency (*p* > 0.10), indicating that mere access to hardware is not sufficient to generate measurable efficiency gains.

## 4. Discussion

### 4.1. Technical Efficiency Performance

The average technical efficiency score of 0.717 suggests that, under prevailing production technologies and managerial conditions, water buffalo farms in the study area were operating below the efficiency frontier, with considerable scope remaining for input optimization.

When compared with findings reported in the national and international literature, the efficiency level observed in this study can be positioned within an intermediate range of the broader empirical distribution. When studies conducted specifically in Türkiye are considered, one study analyzing farms in Iğdır Province reported average technical efficiency scores of 0.84 under constant returns to scale (CRS) and 0.95 under variable returns to scale (VRS) using the input-oriented DEA model [22]. These values were higher than those reported in the current study. These differences may be attributable to variations in production scale, technological adoption levels, access to extension services, and regional market integration.

In contrast, another study examining semi-intensive farms in Balıkesir Province reported pure technical efficiency (VRSTE) of 0.668 and total technical efficiency (CRSTE) of 0.463 in the output-oriented model [24]. Relative to these findings, the performance of farms in the present study may be regarded as comparatively stronger, although still below the frontier observed in more advanced production environments.

Results in the international literature show considerable variation. In a study examining farms in Sri Lanka, the average technical efficiency was reported as 0.868 [19], whereas in another study conducted in the Philippines, this value was determined to be 0.505 using the SFA method [23]. In a further study conducted in the Philippines, overall technical efficiency was estimated at 0.80; however, small-scale farms (0.76) were found to perform worse than commercial farms (0.99) [25]. A recent study of water buffalo farmers in Iraq found the average technical efficiency to be 0.74; this rate increased to 0.78 among those who adopted modern technologies but remained at 0.55 among those who did not [26].

Taken together, these comparisons indicate that measured efficiency outcomes are likely influenced by technological adoption, scale effects, and methodological differences (DEA versus SFA; input- versus output-oriented specifications).

Therefore, the efficiency score of 0.717 achieved in the present study may be interpreted as falling within the upper-middle range of the global distribution reported in the literature while simultaneously reflecting substantial potential for improved resource allocation and productivity enhancement.

### 4.2. Sustainability

The absence of statistically significant differences in economic sustainability across technical efficiency groups suggested that technical efficiency alone may not directly translate into higher economic sustainability performance.

Although efficient farms achieved higher scores in the “relative profit” indicator, their comparatively low performance in “income–expense record-keeping” indicates potential managerial weaknesses. The relatively stronger performance of moderately efficient farms in record-keeping practices appeared to have contributed to their higher composite index scores. This finding highlighted the importance of managerial discipline alongside production efficiency.

Furthermore, the consistently high “forage crop production” index across all efficiency levels suggested that forage crop cultivation has been widely adopted as a standard agricultural practice in the region, independent of technical efficiency status.

Overall, these findings indicated that while technical efficiency enhanced physical production performance, it did not automatically ensure superior economic sustainability outcomes. Managerial capacity, particularly in financial record-keeping and cost management, emerged as a critical complementary factor in strengthening overall economic sustainability.

The absence of statistically significant differences across technical efficiency groups suggested that social sustainability challenges in the region may be structural rather than directly linked to farm-level efficiency performance. The relatively uniform social sustainability scores across groups indicated that broader regional development dynamics may play a determining role.

The paradox between high formal membership in agricultural organizations and zero participation in agricultural extension activities highlighted potential dysfunction within institutional structures. Although membership levels appeared high, the limited provision of technical knowledge and extension services suggested that these organizations may not be effectively fulfilling their developmental role.

The very low levels observed in agricultural extension activities and social activities pointed to systemic weaknesses in knowledge dissemination and social capital formation. The low satisfaction with social life further indicated potential constraints related to rural welfare conditions. Although infrastructure and transportation services were measured at higher levels in efficient farms, the absence of statistical significance suggested that physical infrastructure alone may not be sufficient to ensure stronger social sustainability outcomes.

Overall, while access to veterinary services and family solidarity appeared strong in the region, the inadequacy of extension services and limited social engagement emerged as key vulnerabilities that may threaten the long-term social sustainability of regional water buffalo production systems.

Although an apparent increasing trend across groups was observed, the absence of statistically significant differences suggested that improvements in technical efficiency may not automatically translate into statistically distinguishable environmental performance gains.

The marked discrepancy between awareness-based indicators and implementation-based indicators indicated a structural gap between environmental intentions and actual practice. Despite high awareness scores, the extremely low manure pit index suggested constraints related to infrastructure availability and financial capacity.

The fact that even efficient farms did not reach the “sustainable” threshold (above 0.60) in infrastructure-related indicators supported the interpretation that environmental sustainability challenges may be rooted in regional infrastructure deficiencies rather than purely farm-level managerial differences.

Similarly, while livestock farming is widely perceived as important for biodiversity, the very low “covered space per animal” index highlighted deficiencies in compliance with animal welfare standards.

In conclusion, farms in the region demonstrated relatively high environmental awareness; however, this awareness did not appear to have been fully translated into holistic environmental performance due to limitations in physical infrastructure, particularly manure management systems and shelter conditions.

The overall CSI value of 0.41 placed the production system at the lower bound of the “moderate” sustainability category, suggesting a structurally fragile configuration.

The CSI score obtained in this study was compared with the value of 0.49 reported for cattle breeding in Türkiye [41,51]. In the broader literature, sustainability scores have been reported to range between 0.45 and 0.50 in small-scale operations in India [27,29] and between 37% and 41% in Indonesia and South Africa [28,30].

However, such cross-study comparisons should be interpreted with caution. Differences in indicator selection, weighting schemes, aggregation procedures, sampling frameworks, and even livestock species may limit the methodological comparability of CSI values across studies. Therefore, the numerical similarities or differences reported in the literature should be regarded as indicative rather than strictly equivalent benchmarks.

The finding that the social sustainability dimension emerged as the weakest component was consistent with patterns observed in several small-scale livestock systems in developing countries [30], suggesting that institutional and social constraints may represent a common structural vulnerability.

Furthermore, the absence of statistically significant differences between technical efficiency groups indicated that improvements in production efficiency alone may not be sufficient to generate measurable gains in composite sustainability under existing infrastructural and market conditions.

### 4.3. Determinants of Technical Efficiency

The empirical findings indicated that technical efficiency in water buffalo breeding was systematically related to several farm-level characteristics. The overall pattern of results suggested that efficiency was primarily associated with farmers’ managerial orientation, financial conditions, and access to information. In particular, the significance and direction of the experience, income, indebtedness, and digital variables revealed that both managerial allocation and financial capacity play decisive roles in shaping technical performance.

The negative and statistically significant effect of crop production experience indicated that as farmers concentrate more on crop production, the labor, time, and managerial attention allocated to buffalo breeding may decrease. This result suggested that greater engagement in crop production may reduce the managerial attention and resource allocation devoted to buffalo breeding, thereby lowering branch-specific technical efficiency within the farm. In contrast, experience in livestock breeding exerted a positive and statistically significant effect on technical efficiency. This finding implied that accumulated herd management knowledge, husbandry skills, and sector-specific experience enhanced production performance. The cumulative effect of practical livestock experience appeared to be more decisive for improving technical efficiency than experience acquired in other agricultural activities.

The positive and statistically significant coefficient of non-farm income suggested that alternative income sources contributed positively to technical efficiency. Non-farm income may ease cash-flow constraints, enable timely procurement of feed and other inputs, and improve farmers’ ability to manage market risks. In this context, income diversification appeared to function as a stabilizing mechanism that supports operational efficiency under volatile economic conditions.

One of the most striking findings concerns the financial structure of farms. The negative and statistically significant effect of indebtedness status indicated that indebted farmers tended to exhibit lower technical efficiency compared to non-indebted farmers. Debt obligations may constrain managerial flexibility, distort optimal input combinations, and reduce farmers’ ability to allocate resources efficiently, ultimately lowering technical efficiency levels.

With regard to digital factors, internet access positively and significantly influenced technical efficiency, indicating that connectivity enhanced access to market information, extension services, and technical knowledge. However, computer ownership did not show a statistically significant effect, suggesting that infrastructure alone did not guarantee productivity gains unless it was effectively utilized for production- and management-related purposes. These results implied that functional access to information networks was more critical than mere ownership of technological equipment. Nevertheless, the positive association between internet access and technical efficiency should be interpreted with caution. It is possible that more efficient farms are also more likely to adopt digital tools, suggesting potential reverse causality. Moreover, internet access may proxy for unobserved factors such as farm scale, market integration, or managerial openness to innovation. Therefore, the results should be viewed as associative rather than strictly causal.

Finally, although the coefficient of education level was negative and only marginally significant, the results indicate that formal schooling did not produce a statistically meaningful improvement in technical efficiency among buffalo farmers. This finding partially contradicts previous studies reporting a positive association between education and farm performance [5,24]. However, in the present sample, the limited variation in formal education levels and the dominance of traditional production practices may explain why formal schooling does not translate into measurable efficiency gains. In traditional buffalo breeding systems, experiential knowledge and practical expertise appear to outweigh formal educational attainment in shaping production performance.

The findings revealed that technical efficiency in water buffalo breeding was primarily shaped by sector-specific experience, financial resilience, and effective access to information rather than by general demographic characteristics. While some results were consistent with the existing literature, others highlighted context-specific dynamics that differentiate buffalo breeding farms from other livestock production systems. These outcomes underscored the importance of managerial specialization, balanced financial structures, and functional digital access in enhancing efficiency within traditional buffalo farming environments.

### 4.4. Limitations and Future Directions

Several limitations of this study should be acknowledged. First, the empirical analysis was limited to a single province, which may constrain the generalizability of the findings to other water buffalo farming regions characterized by different structural, institutional, and market conditions. Second, the cross-sectional design restricts the ability to establish causal relationships among the variables examined; therefore, the findings should be interpreted as associative rather than causal. In addition, the relatively limited variation in formal education levels within the sample and potential measurement constraints regarding educational attainment may have reduced the statistical power to detect a clearer effect of education on technical efficiency. Third, due to data constraints, the environmental dimension does not include direct indicators of greenhouse gas emissions or water-use intensity, which are increasingly recognized as critical components of livestock sustainability assessments.

Building on these limitations, future research could adopt longitudinal designs and panel data approaches across different provinces in Türkiye to examine dynamic changes in sustainability performance and institutional evolution over time. Comparative analyses across Mediterranean water buffalo farming regions may also provide deeper insights into how diverse policy frameworks and market structures influence sustainability outcomes. Moreover, integrating additional environmental indicators—such as greenhouse gas emissions and water footprint measures—would further strengthen the robustness and external validity of composite sustainability assessments in traditional, low-input livestock systems.

## 5. Conclusions

The empirical findings indicate that the average technical efficiency score was 0.717, while the Composite Sustainability Index (CSI) averaged 0.41. Moreover, no statistically significant relationship was identified between technical efficiency and composite sustainability performance. This study demonstrated that while achieving technical efficiency in water buffalo breeding provided the basis for a sustainable production model, it was insufficient on its own under current market and infrastructure conditions. The farms in the research area exhibited a fragile structure, remaining at the lower end of the “moderate” level with a Composite Sustainability Index. More importantly, contrary to the theoretical assumption that increasing technical efficiency at the farm level would automatically improve sustainability performance, a structural disconnect between technical efficiency and sustainability performance was found in regional water buffalo breeding. The inability of even the most efficient farms to differentiate themselves from other groups in terms of financial record-keeping, organizational strength, and environmental investment suggested that the problem stemmed from macro-level structural bottlenecks rather than micro-level management errors. The analysis revealed two fundamental structural vulnerabilities specific to the region. Firstly, there was a substantial gap between environmental intentions and concrete practices. Although farmers were found to have a high level of environmental awareness, insufficient capital accumulation and deficient infrastructure prevented this awareness from translating into investments such as manure management. The second issue is dysfunctional organization: despite seemingly high cooperative rates on paper, the inability to provide farmers with technical information and to disseminate knowledge was identified as the most critical factor weakening social sustainability. Furthermore, it was determined that using digital tools solely for social interaction, rather than as sources of agricultural information, created information asymmetry that reduces productivity. Conversely, external financing sources were a critical solution for overcoming input constraints. For the sector to escape its current “fragile middle” status and achieve lasting sustainability, it is deemed essential for policymakers to shift from quantity-focused support to a structural transformation-focused model. While current quantity-focused support (per-animal payments, etc.) encouraged farms to increase physical capacity, its failure to reward sustainability criteria such as record-keeping, waste management, and professional organization deepened this structural disconnect. At the national level, existing support schemes primarily emphasize production and capacity expansion, reinforcing the need to integrate explicit sustainability criteria into public support frameworks. In this context, we recommend that policymakers shift from quantity-focused support mechanisms toward a structural transformation model that transforms agricultural organizations into active extension centers that provide financial literacy and technical knowledge for farmers and direct public support towards modernization projects that translate environmental awareness into action. These policy implications are directly grounded in the empirical findings, particularly the absence of a statistically significant relationship between efficiency and sustainability, the negative association of indebtedness with technical efficiency, and the identified gaps in environmental and organizational performance.

Finally, although the empirical findings of this study are specific to the regional water buffalo production system examined, the structural patterns identified—such as institutional weaknesses, limited financial literacy, gaps between environmental awareness and implementation, and information asymmetry within producer organizations—are not unique to this context. Similar structural constraints are observed in other small- and medium-scale water buffalo farming regions in Türkiye as well as in traditional, low-input livestock systems operating under comparable institutional and infrastructural conditions. However, since the empirical evidence is derived from a single province, strict national-level generalization should be approached with caution. Nevertheless, the findings may provide indicative insights for regions facing similar structural, institutional, and market conditions.

## Figures and Tables

**Table 1:** Indicators of economic sustainability.

Indicator	Unit/Measurement	Expected Sign *
Gross value of production	$ Farms^−1^	+
Relative profit	$ Livestock Units^−1^	+
Absolute profit	$ Livestock Units^−1^	+
Livestock units	$ Livestock Units^−1^	−
Production cost	Livestock Units	+
Yield	Liter Head^−1^	+
Lactation period	Days	+
Number of animal deaths	Head Farms^−1^	−
Forage crop production	Binary (No = 0 Yes = 1)	+
Savings status	Binary (No = 0 Yes = 1)	+
Indoor feeding period	Days	−
Record-keeping status	Binary (No = 0 Yes = 1)	+
Pasture feeding	Binary (No = 0 Yes = 1)	+

* The Unit/Measurement column describes the scale or physical unit used for each indicator. Expected Sign indicates the hypothesized direction of each indicator’s contribution to the Sustainability Index: ‘+’ denotes a positive contribution, and ‘−’ denotes a negative contribution.

**Table 2:** Social sustainability indicators.

Indicator	Unit/Measurement *	Expected Sign **
Age of the farm manager	Years	−
Duration of education	Years	+
Years of experience	Years	+
Communication with institutions	Frequency (1–6)	+
Participation in extension activities	Frequency (1–6)	+
Agricultural organization membership	Binary (Yes = 1, No = 0)	+
Satisfaction with social life	Binary (Yes = 1, No = 0)	+
Engagement in social activities	Binary (Yes = 1, No = 0)	+
Access to healthcare services	Binary (Yes = 1, No = 0)	+
Access to education services	Binary (Yes = 1, No = 0)	+
Adequacy of transport & infrastructure	Binary (Yes = 1, No = 0)	+
Access to veterinary & health services	Binary (Yes = 1, No = 0)	+
Satisfaction with farming activities	Likert scale (1–5)	+
Interest level in farming activities		+
Frequency of using communication tools	Frequency (1–6)	+
Importance of family members’ opinions	Likert scale (1–5)	+
Importance of family contribution		+
Willingness of family to continue water buffalo breeding	Binary (Yes = 1, No = 0)	+

* The Unit/Measurement defines the data structure used for each indicator. Binary variables are coded as 1 for ‘Yes’ and 0 for ‘No’. Frequency scales (1–6) range from “Never” to “More than four times a year”. Likert scales (1–5) range from ‘Strongly Disagree’ to ‘Strongly Agree’. ** Expected Sign indicates the hypothesized direction of the indicator’s contribution to the Social Sustainability Index (+ for positive, − for negative).

**Table 3:** Environmental sustainability indicators.

Indicator	Unit/Measurement	Expected Sign *
Adequacy of pasture areas	Binary (Yes = 1 No = 0)	+
Recent reduction in pasture areas	Yes = 0 No = 1	−
Adequacy of pasture grass quality	Yes = 1 No = 0	+
Negative impact of current pasture status on water buffalo numbers	Yes = 0 No = 1	−
Perception of water buffalo breeding as important for biodiversity	Binary (Yes = 1 No = 0)	+
Commitment to environmentally friendly practices in water buffalo breeding	Binary (Yes = 1 No = 0)	+
Importance given to the hygiene of tools and machinery	Binary (Yes = 1 No = 0)	+
Indoor area per water buffalo	Square meter	+
Utilization of manure as fuel	Binary (Yes = 0 No = 1)	−
Adequacy of shelter infrastructure	Binary (Yes = 1 No = 0)	+
Presence of other cattle breeding	Binary (Yes = 0 No = 1)	−
Availability of manure pits	Binary (Yes = 1 No = 0)	+

* Expected Sign indicates the hypothesized direction of the indicator’s contribution to the Sustainability Index: (+) denotes a positive contribution, while (−) denotes a negative contribution.

**Table 4:** Summary statistics for the variables used in the efficiency analysis.

Variables	Minimum	Maximum	Mean	S.D.
DEA model				
Output				
Gross Production Value ($ farm^−1^)	3015.194	52,692.719	13,800.569	9760.544
Inputs				
Roughage (kg farm^−1^)	2657.600	445,720.000	39,833.970	73,105.214
Concentrate (kg farm^−1^)	0.010	110,000.000	13,151.112	20,132.444
Water buffalo (LU)	4.500	83.560	22.365	18.438
Labor (h farm^−1^)	34.800	5690.000	1614.254	1304.088
Veterinary costs ($ farm^−1^)	0.000	1533.386	509.967	323.661

**Table 5:** Frequency distributions of efficiency scores obtained with the DEA model.

Efficiency Score Range	Farm (N)	Percentage (%)
0.75–1.00	31	43.06
0.51–0.74	20	27.78
<0.50	21	29.17
**Total**	**72**	**100.00**
**Summary Statistics**		
**Mean**	**0.717**	
Minimum	0.221	
Maximum	1.000	
Std. Deviation	0.236	

**Table 6:** Results of the Economic Sustainability Index.

Indicator	Inefficient		Moderately Efficient		Efficient		Farms	
	Mean	S.D. *	Mean	S.D.	Mean	S.D.	Mean	S.D.
Gross value of production	0.15	0.16	0.23	0.22	0.26	0.20	0.22	0.20
Relative profit	0.16	0.13	0.29	0.15	0.40	0.27	0.30	0.22
Absolute profit	0.18	0.13	0.30	0.15	0.36	0.21	0.29	0.19
Livestock units	0.25	0.29	0.23	0.21	0.20	0.21	0.23	0.23
Production cost	0.67	0.32	0.75	0.24	0.71	0.29	0.71	0.28
Yield	0.38	0.20	0.36	0.20	0.35	0.14	0.36	0.18
Lactation period	0.31	0.45	0.32	0.47	0.24	0.37	0.28	0.42
Number of animal deaths	0.38	0.50	0.40	0.50	0.35	0.66	0.38	0.57
Forage crop production	0.90	0.30	0.90	0.31	0.90	0.30	0.90	0.30
Savings status	0.33	0.48	0.30	0.47	0.32	0.48	0.32	0.47
Indoor feeding period	0.68	0.45	0.90	0.30	0.91	0.26	0.84	0.35
Record-keeping status	0.14	0.36	0.30	0.47	0.10	0.30	0.17	0.38
Pasture feeding	0.61	0.40	0.79	0.27	0.82	0.22	0.75	0.31
**Economic Sustainability Index**	**0.43**	0.17	**0.47**	0.10	**0.46**	0.13	**0.45**	0.13

* Std. Deviation.

**Table 7:** Social Sustainability Index results.

Indicators	Inefficient		Moderately Efficient		Efficient		Farms	
	Mean	S.D.	Mean	S.D.	Mean	S.D.	Mean	S.D.
Age of the farm manager	0.36	0.32	0.33	0.22	0.37	0.22	0.35	0.25
Duration of education	0.58	0.18	0.58	0.24	0.59	0.20	0.58	0.21
Years of experience	0.51	0.42	0.46	0.33	0.52	0.34	0.50	0.36
Communication with institutions	0.51	0.11	0.48	0.01	0.47	0.12	0.49	0.10
Participation in extension activities	0.00	0.00	0.05	0.15	0.06	0.21	0.04	0.16
Agricultural organization membership	1.00	0.00	0.95	0.22	0.90	0.30	0.94	0.23
Satisfaction with social life	0.14	0.36	0.15	0.37	0.19	0.40	0.17	0.38
Engagement in social activities	0.10	0.30	0.00	0.00	0.10	0.30	0.07	0.26
Access to healthcare services	0.71	0.46	0.90	0.31	0.81	0.40	0.81	0.40
Access to education services	0.57	0.51	0.80	0.41	0.68	0.48	0.68	0.47
Adequacy of transport & infrastructure	0.67	0.48	0.75	0.44	0.84	0.37	0.76	0.43
Access to veterinary & health services	1.00	0.00	0.95	0.22	1.00	0.00	0.99	0.12
Satisfaction with farming activities	0.63	0.22	0.56	0.16	0.68	0.22	0.63	0.21
Interest level in farming activities	0.59	0.31	0.42	0.30	0.62	0.38	0.56	0.35
Frequency of using communication tools	0.33	0.18	0.32	0.10	0.29	0.12	0.31	0.13
Importance of family members’ opinions	0.95	0.12	0.97	0.10	0.91	0.21	0.94	0.16
Importance of the family’s contribution	0.68	0.07	0.67	0.00	0.69	0.08	0.68	0.07
Willingness of the family to continue water buffalo breeding	0.24	0.44	0.15	0.37	0.19	0.40	0.19	0.40
**Social Sustainability Index**	**0.43**	0.07	**0.43**	0.05	**0.45**	0.06	**0.44**	0.06

**Table 8:** Environmental Sustainability Index results.

Indicators	Inefficient		Moderately Efficient		Efficient		Farms	
	Mean	S.D.	Mean	S.D.	Mean	S.D.	Mean	S.D.
Adequacy of pasture areas	0.38	0.50	0.40	0.50	0.52	0.51	0.44	0.50
Recent reduction in pasture areas	0.38	0.50	0.45	0.51	0.52	0.51	0.46	0.50
Adequacy of pasture grass quality	0.43	0.51	0.45	0.51	0.52	0.51	0.47	0.50
Negative impact of the current pasture status on water buffalo numbers	0.19	0.40	0.30	0.47	0.32	0.48	0.28	0.45
Perception of water buffalo breeding as important for biodiversity	0.81	0.40	0.75	0.44	0.77	0.43	0.78	0.42
Commitment to environmentally friendly practices in water buffalo breeding	0.95	0.22	1.00	0.00	0.97	0.18	0.97	0.17
Importance given to the hygiene of tools and machinery	0.95	0.22	1.00	0.00	1.00	0.00	0.99	0.12
Indoor area per water buffalo	0.06	0.05	0.12	0.23	0.06	0.07	0.08	0.13
Utilization of manure as fuel	0.10	0.30	0.05	0.22	0.06	0.25	0.07	0.26
Adequacy of shelter infrastructure	0.57	0.08	0.59	0.07	0.59	0.14	0.58	0.11
Presence of other cattle breeding	0.71	0.46	0.75	0.44	0.71	0.46	0.72	0.45
Availability of manure pits	0.05	0.22	0.05	0.22	0.29	0.46	0.15	0.36
**Environmental Sustainability Index**	**0.47**	0.17	**0.49**	0.19	**0.53**	0.18	**0.50**	0.18

**Table 9:** Comparative analysis of sustainability dimensions and Composite Sustainability Index across different efficiency groups.

Dimensions	Inefficient		Moderately Efficient		Efficient		Farms	
	Mean	S.D.	Mean	S.D.	Mean	S.D.	Mean	S.D.
Economic	0.43	0.17	0.47	0.10	0.46	0.13	**0.45**	0.13
Social	0.43	0.07	0.43	0.05	0.45	0.06	**0.44**	0.06
Environmental	0.47	0.17	0.49	0.19	0.53	0.18	**0.50**	0.18
**Composite Sustainability index**	**0.37**	0.11	**0.38**	0.09	**0.45**	0.17	**0.41**	0.14

**Table 10:** Kruskal–Wallis H test results across efficiency groups.

Dimensions	H Statistic	df	*p*-Value
Economic	2.577	2	0.276
Social	2.384	2	0.304
Environmental	2.153	2	0.341
Composite Sustainability index	4.548	2	0.103

**Table 11:** Summary statistics for variables used in Tobit analysis.

Variables	Minimum	Maximum	Mean	S.D.
Experience in plant production (year)	0.000	51.000	32.750	12.683
Non-farm income (1: Yes, 0: No)	0.000	1.000	0.486	0.503
Experience in livestock breeding (year)	5.000	55.000	32.458	13.457
Education level (0: No Formal Education, 1: Primary School, 2: Middle School, 3: High School, 4: Associate’s Degree, 5: Bachelor’s Degree)	0.000	5.000	2.556	0.963
Indebtedness status (1: Yes, 0: No)	0.000	1.000	0.708	0.458
Availability of a computer (1: Yes, 0: No)	0.000	1.000	0.625	0.488
Availability of an internet connection (1: Yes, 0: No)	0.000	1.000	0.750	0.436

**Table 12:** The parameters and their standard errors of the Tobit model.

Variable	Coefficient	Std. Error	z-Score	Significance	AME *
**Constant (c)**	**0.803**	**0.153**	**5.239**	**0.000**	**-**
Experience in plant production	−0.014	0.004	−3.123	0.002	−0.011
Non-farm income	0.255	0.067	3.815	0.000	0.206
Experience in livestock breeding	0.016	0.004	3.703	0.000	0.013
Education level	−0.059	0.032	−1.858	0.063	−0.047
Indebtedness status	−0.203	0.066	−3.083	0.002	−0.164
Availability of a computer	−0.118	0.078	−1.510	0.131	−0.095
Availability of an internet connection	0.176	0.088	2.010	0.044	0.142
Number of observations = 72 Uncensored observations = 58 Right-censored observations = 14 Log-likelihood = −8.599 LR χ^2^(7) = 29.23 (*p* < 0.001)					

* The dependent variable is the technical efficiency score (0–1). AME denotes the average marginal effects on the observed efficiency score.

## Data Availability

The datasets generated and analyzed during the current study are not publicly available due to privacy and ethical restrictions, as the data were collected from individual farmers under written informed consent and ethical approval. Requests for access to the data may be directed to the corresponding author, subject to ethical considerations.

## Abbreviations

The following abbreviations are used in this manuscript:

DEA	Data Envelopment Analysis
AHP	Analytic Hierarchy Process
CI	Consistency Index
CR	Consistency Ratio
MDS	Multidimensional Scaling
MESMIS	Framework for Assessing the Sustainability of Natural Resource Management Systems using Sustainability Indicators
SAFA	Sustainability Assessment of Food and Agriculture Systems
RAP	Rapid Appraisal for Sustainability
VRS	variable Returns to Scale
GPV	Gross Production Value
LU	Livestock Units
CSI	Composite Sustainability Index
DEAP	Data Envelopment Analysis Program
ESI	Economic Sustainability Index
SSI	Social Sustainability Index
EnSI	Environmental Sustainability Index
TE	Technical Efficiency
CRS	Constant Returns to Scale
VRSTE	Pure Technical Efficiency
SFA	Stochastic Frontier Analysis
S.D.	Std. Deviation
AME	Average Marginal Effects
